# 16S rDNA sequencing analyzes differences in intestinal flora of human immunodeficiency virus (HIV) patients and association with immune activation

**DOI:** 10.1080/21655979.2021.2019174

**Published:** 2022-02-06

**Authors:** Zhang Mingjun, Mo Fei, Xu Zhousong, Xu Wei, Xu Jian, Yi Yuanxue, Shen Youfeng, Chen Zhongping, Long Yiqin, Zhao Xiaohong, Cheng Ying, Wang Zhenbing, Deng Zehu, Li Lanjuan

**Affiliations:** a State Key Laboratory for Diagnosis and Treatment of Infectious Diseases, National Clinical Research Center for Infectious Diseases, National Medical Center for Infectious Diseases, Collaborative Innovation Center for Diagnosis and Treatment of Infectious Diseases, The First Affiliated Hospital, Zhejiang University School of Medicine, Hangzhou, China; b Department of Laboratory Medicine, People’s Hospital of Jiulongpo District, Chongqing, China; c Department of Laboratory Medicine, Hangzhou Shulan Hospital, Zhejiang University, Hangzhou, China; d Department of Laboratory Medicine, Hangzhou Tongchuang Medical Laboratory Co. LTD, Hangzhou, China; e Department of Laboratory Medicine, Chongqing Precision Medical Industry Technology Research Institute, Chongqing, China; f Department of Laboratory Medicine, Chongqing D.A. Medical Laboratory, Chongqing, China

**Keywords:** 16s rDNA, AIDS, HIV, intestinal flora

## Abstract

To clarify the influence of HIV on the intestinal flora and the interrelationship with CD4 T cells, the present study collected stool specimens from 33 HIV patients and 28 healthy subjects to compare the differences in the intestinal flora and CD4 T cells in a 16S rDNA-sequencing approach. ELISA was used to detect the expressions of interleukin 2 (IL-2), IL-8, and tumor necrosis factor-α (TNF-α). Meanwhile, correlation analysis with the different bacterial populations in each group was carried out. The results revealed that Alpha diversity indices of the intestinal flora of HIV patients were markedly lower than that of the healthy group (p < 0.05). The top five bacterial species in the HIV group were Bacteroides (23.453%), Prevotella (19.237%), Fusobacterium (12.408%), Lachnospira (3.811%), and Escherichia-Shigella (3.126%). Spearman correlation analysis results indicated that Fusobacterium_mortiferum, Fusobacterium, and Gammaproteobacteria were positively correlated with TNF-α (p < 0.05), whereas Ruminococcaceae, Bacteroidales was negatively correlated with TNF-α (p < 0.05). Additionally, Agathobacter was positively correlated with contents of IL-2 and IL-8 (p < 0.05), whereas Prevotellaceae, and Prevotella were negatively correlated with IL-8 content (p < 0.05). Furthermore, the top five strains in the CD4 high group (≥350/mm^3^) included Bacteroides (23.286%), Prevotella (21.943%), Fusobacterium (10.479%), Lachnospira (4.465%), and un_f_Lachnospiraceae (2.786%). Taken together, the present study identified that Fusobacterium and Escherichia-Shigella were specific and highly abundant in the HIV group and a correlation between the different bacterial flora and the contents of IL-2, IL-8, and TNF-α was revealed.

## 1. Introduction

Acquired immunodeficiency syndrome (AIDS) is an immunodeficiency disease caused by HIV and has attracted the attention of many researchers of its high infection and incurable nature. AIDS has emerged as a public health problem that seriously threatens people’s health in China, and it has also impeded economic development and social stability [1]. As a chronic RNA virus, the pathogenic mechanism of HIV is to attack the helper T lymphocyte, CD4 cell, causing damage to the body’s immune functions and various opportunistic infections. Meanwhile, the intestine is enriched with lymphatic tissues, lymph nodes, and lymphocytes [2]. Some studies have revealed that the intestine is an important part of HIV infection, accumulating a great deal of HIV **[**3**]** and making the real virus reservoir of AIDS victims. Nearly 80% of HIV hides in the intestinal tissue, whereas the viral load in the blood accounts for only 2%–5%. As the major targets attacked by HIV are lymphocytes after the invasion of the human body, most HIV-infected patients develop possible gastrointestinal symptoms of diarrhea, nausea, vomiting, and abdominal pain, among which diarrhea is the most common one. Diarrhea is the result of unbalanced intestinal microecology, which leads to endogenous infection in immunocompromised patients. Furthermore, it directly promotes the activation of the immune system in chronic HIV infection, which ultimately determines the progress of AIDS **[**4**]**. The influencing factors imbalance of the intestinal immune barrier, the translocation of immune-stimulating microbial products, and chronic systemic inflammation are responsible for the progression of the disease to AIDS. Intestinal microecology balance helps to fortify the immunity of the human body.

Recent studies have shown that AIDS morbidity develops because 80% of HIV first accumulates in the intestinal wall, directly consuming a large number of CD4 cells **[**5**]**. Meanwhile, HIV directly destroys the integrity of the intestinal epithelial lining, making it easier for microbes normally present in the gut lumen to enter the lamina propria. And the translocation and transference of intestinal flora cause direct depletion of some effector cells (Th22 and T17/Treg) that are sensitive to specific bacteria, allowing some inflammatory factors IL-10, IL-22, and IL-6 and bacterial metabolites LPS to enter the blood circulation directly. Consequently, it initiates downstream immune activation and causes local and systemic inflammation, thereby continuously consuming CD4 cells and concurrent symbiosis opportunistic infections of bacteria and other diseases **[**6**]**. Other research has found that T cell subsets (repair T cells Th22, pro-inflammatory cells Th17, and immune regulatory T cells Tregs) are closely related to the proportion of symbiotic bacteria and translocation of microorganisms **[**7**]- [**8,9**]**. The previously described cells mainly secrete cytokines IL-4, IL-10, and TGF-β, and participate in clinical symptom improvement of multiple immune diseases, including autoimmune diseases, transplantation immunity, and tumors **[**10**]**.

However, little research has been conducted to investigate the relationship between intestinal flora and CD4 T cells and inflammatory factors in HIV patients. 16S rDNA identification is to identify bacterial species by using bacterial 16S rDNA sequencing. This technique is the most useful and commonly applied molecular clock in the systematic taxonomy of bacteria as 16S rDNA has only a few types, large content, moderate molecular size, and exists in all organisms. The size of 16S rDNA is about 1.5 Kb, which can not only reflect the differences between various bacterial genera but also can easily obtain its sequence using sequencing technology. We assumed that the intestinal flora of HIV patients differed from that of the normal population, and might be related to the expressions of inflammatory factors. We attempted to analyze the differences in intestinal flora to adjust the balance of the intestinal microecology of HIV patients by supplementing certain specific probiotics in the future and finding a supplementary treatment method for HIV patients to delay the onset of AIDS or cure them. Therefore, this study collected stool specimens from HIV patients and healthy individuals, performed 16S rDNA sequencing to compare the differences of bacterial populations between the HIV and healthy groups, the high and the low expression CD4 T cell groups. Additionally, the contents of IL-2, IL-8, and TNF-α of each sample were obtained for correlation analysis of different bacterial groups. The current study clarified the intestinal microecology and immune activation of T cell subsets in HIV patients, which served as a research basis for finding ways of HIV treatment and postponing the onset of AIDS.

## 2. Materials and methods

### 2.1. Clinical data

The present research was a prospective and observational study and approved by the Medical Ethics Committee of the People’s Hospital of Jiulongpo District, Chongqing (2019 Ethical Review No. 01). HIV patients and healthy individuals (HIV spared) were recruited from the People’s Hospital of Jiulongpo District, Chongqing. The samples were collected from September 11 to 31 December 2020. Inclusion criteria for HIV patients included those infected with HIV-1 virus, those with positive chemiluminescence HIV test results, and those with abnormal baseline liver function, kidney function, blood lipids, blood sugar, and thyroid function. Exclusion criteria were subjects with anemia or decreased blood cells, thrombocytopenia, tumors, liver function decompensation, portal hypertension, clinical hepatitis, and other causes of colitis, not caused by HIV. Inclusion criteria for healthy individuals were those who do not smoke or drink, those with normal stool and had no diarrhea; No hepatitis, tuberculosis, coronary heart disease, tumor, and other underlying diseases; Laboratory tests of liver function, kidney function, blood lipids, blood sugar, and thyroid function were all normal. The qualified samples were ultimately collected from 33 HIV patients and 28 healthy individuals.

### 2.2. Blood sample collection and serological testing

Five milliliters of fasting blood samples were collected in a vacuum blood tube, kept at room temperature for 30 min, centrifuged at 3000 g for 10 min, frozen at −80°C, and placed at room temperature for remelting before use. Four milliliters of anticoagulant EDTA-K2 blood were collected and detected within 8 h by flow cytometry.

Cytokine levels of IL-2, IL-8, and TNF-α in the serum were measured by a quantitative sandwich enzyme-linked immunosorbent assay (ELISA), with a Quantikine kit (Shanghai Kehua, CHINA). Sample absorbance was determined using a microplate reader, and data were analyzed using the ELISACal software. Each sample was measured 3 times and the mean value was obtained.

The absolute counts (cells/μl) of mature human helper/inducer (CD4+) T lymphocytes and suppressor/cytotoxic (CD8+) T lymphocytes in erythrocytosed whole blood were determined using CD4/CD8 kits (Celula, China) [11,12].

### 2.3 Collection of stool specimens

The sample collection procedures were strictly performed following the aseptic operation requirements. Sterile cotton swabs were applied to peel off the surface of fresh feces to avoid mixing urine or other debris and replaced to pick at least 1 g of the inner feces and put into a sterile cryotube. Immediately the specimens were stored in a refrigerator at −20°C, transferred to the laboratory within 24 h, and kept in a refrigerator at −80°C for later use.

### 2.4. DNA extraction and purification of intestinal flora

QlAamp DNA Stool Mini Kit extraction kit (Qiagen, Germany) was employed to extract DNA, and QubitFluorometer kit (Life Technologies, USA) was adopted to detect DNA concentration, and 1% agarose gel electrophoresis was added to detect sample integrity. Taq enzyme (Shanghai Shenggong) was used to amplify the selected region (V3~ V4 regions), PCR primers were 338 F (5ʹ-ACTCCTACGGGAGGCAGCA-3ʹ) and 806B (5ʹ-GGACTACHVGGGTWTCTAAT-3ʹ). After passing the 1% agarose gel electrophoresis test, the samples were delivered for 16S high-throughput sequencing analysis of intestinal bacteria in the laboratory.

### 2.5. 16S high-throughput sequencing of bacteria

Ilumina Hiseq 2500 (Ilumina, USA) sequencing platform was employed for PE250 library construction and sequencing, using a pair-end sequencing (Pair-end) method. Each sequence generated 250 reads from the 5ʹ end to the 3ʹ end. The original data obtained from sequencing were sheared and filtered before analysis to rule out low-quality reads and valid data were obtained. The reads were spliced into tags through the overlapping relationship between the valid data, and further filtering helped to obtain the target fragments. Based on 97% similarity, the tags were clustered into operational taxa (OTU), compared with the 16S database of known species, and annotated the OTU species, thereby obtaining the community composition of each sample [13].

### 2.6. Intestinal flora analysis method

QIIME2 software (Version 2.0.2, https://qiime2.org/) was used to analyze the microbiome sequence data [14]. The 16S rRNA representative sequence was constructed using the silva-138-99 (QIIME 2 2020.8 Species Annotation Database) provided by SILVA (https://www.arb-silva.de/) amplicon database with a similarity threshold of 97%. To remove the potential signals in the contaminants, we removed the analytical taxa with a relative abundance of 10% and the OTUs with a total abundance of 1 in the sample from the analytical taxa. The remaining reads were aggregated into 3942 OTUs.

Alpha diversity used observed species index (actual number of observed OTUs), Chao1 index (estimated total number of OTUs in the sample), ACE index (estimated total number of OTUs contained in the sample), Shannon index (estimated microbial community diversity), and Simpson index (estimated microbial community diversity). The Alpha diversity of the microbial community in the HIV group and the Norma group was analyzed. Pie charts and histograms were plotted to present the proportion of genus level of microorganisms, which was the relative abundance in the sample. Linear discriminant analysis effect size (LEfSe) could discover biomarkers and determine the best characteristic genus for each experimental group. The LEfSe scores measured the consistency of the relative abundance differences of taxa in each group. The higher the score, the higher the consistency [15]. The groups with LDA scores > 4 and P < 0.05 were considered significant. The differential species at the genus level and the provided physical and chemical factors were employed to calculate the Spearman correlation between the two. R language (4.1.0, https://www.r-project.org/) software psych package/reshape2 package/ggplot2 package was adopted to plot heat maps and the vital relationships between different species and physical and chemical factors were revealed [16].

### 2.7. Statistical analysis

SPSS 22.0 statistical analysis software was employed for data analysis, and the measurement index was expressed as x ± s. T tests were adopted for the data conformed to normal distribution. Categorical count indexes were compared using χ^2^ tests or Fisher’s exact probability method. Hypothesis tests were performed using two-sided testing to obtain the measurements and the corresponding P values. P < 0.05 was considered as the standard of significant difference.

## 3. Results

To clarify the impact of HIV on the intestinal flora and its relationship with CD4 T cells, this study adopted 16S rDNA sequencing to compare the differences in intestinal flora in stool samples collected from HIV patients and normal people. ELISA was used to detect the expressions of IL-2, IL-8, and TNF-α in the serum of samples, and the correlation analysis with the different bacterial groups in each group was carried out. We analyzed the difference in the intestinal flora and provided a potential research basis for the future treatment of AIDS by supplementing certain specific probiotics, hoping to adjust the balance of the intestinal microecology of HIV patients.

### 3.1. Differential analysis of intestinal flora between HIV patients and healthy individuals

**Figure 1.**
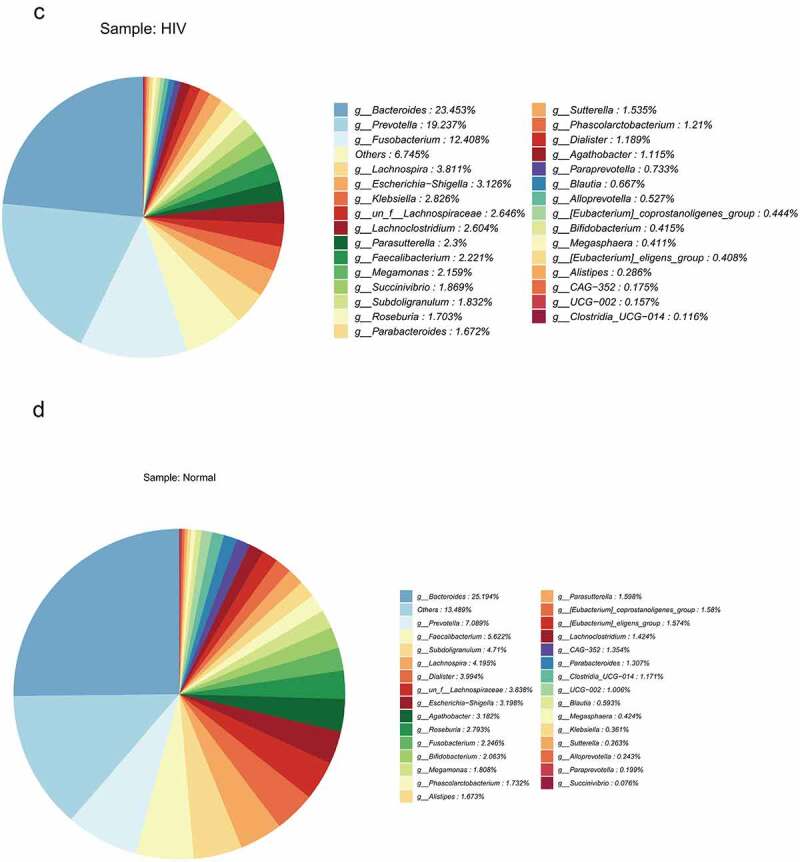
Continued.

In this study, 16S sequencing was performed to identify stool specimens obtained from 33 HIV patients and 28 healthy humans. A total of 4,865,329 reads and 3 942 OTUs were obtained by clustering from the samples. QIIME software was utilized to calculate alpha diversity indices in the samples of both groups. The findings revealed that the observed species index, Chao1 index, ACE index, Shannon index, and Simpson index of the HIV group were markedly lower than those of the healthy group (p < 0.05) (Figure 1(a)).**Figure 1.**
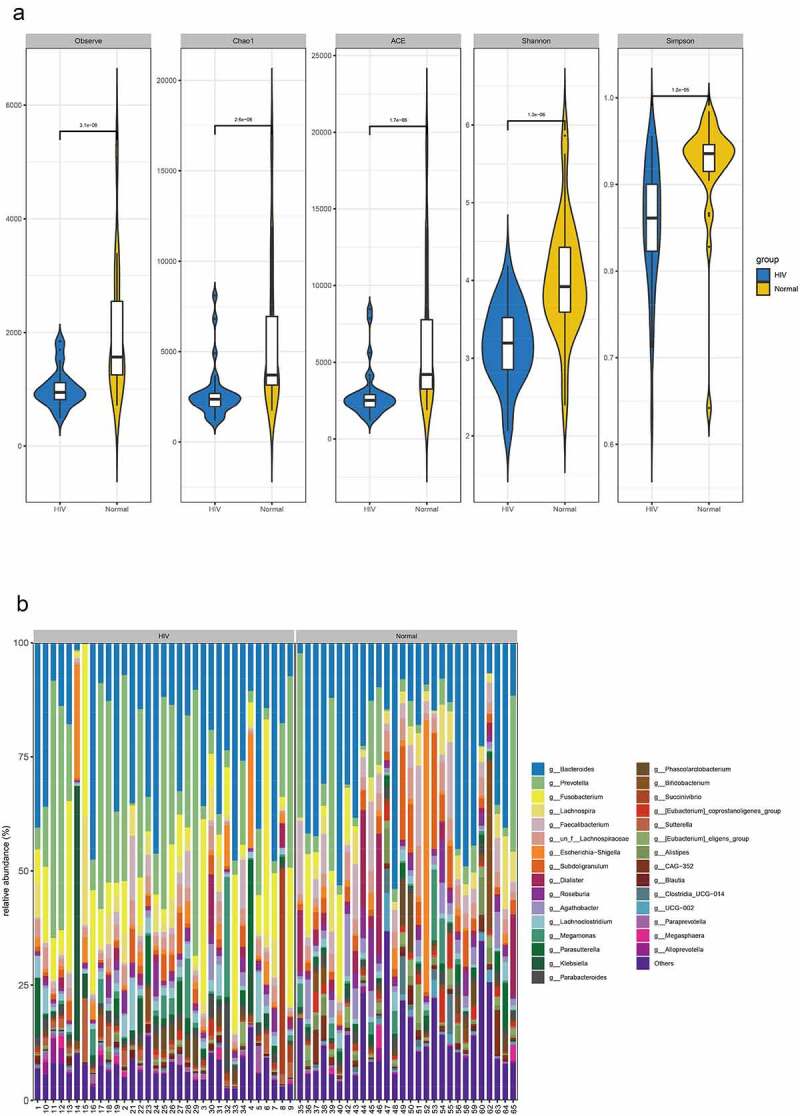
Differential analysis of intestinal flora between HIV patients and healthy individuals. A Alpha diversity analysis of OTU number and microbial colony diversity in both groups. Observed species indices (the actual number of OTU on observation), Chao1 index (estimated total number of OTU contained in the samples), ACE index (estimated total number of OTU contained in the samples), Shannon index (estimated microbial colony diversity), Simpson index (estimated microbial colony diversity). B, Cluster analysis of flora. C, Statistical results of bacterial abundance in the HIV group. D, Statistical results of bacterial abundance in the healthy group.

Relative abundance and species clustering of dominant intestinal flora were compared at genus level between both groups, and the findings suggested that the top five bacterial species in the HIV group were Bacteroides (23.453%), Prevotella (19.237%), Fusobacterium (12.408%), Lachnospira (3.811%) and Escherichia-Shigella (3.126%) (Figure 1(b,c)). The top five bacterial species in the healthy group were Bacteroides (25.194%), Prevotella (7.089%), Faecalibacterium (5.622%), Subdoligranulum (4.71%), and Lachnospira (4.195%) (Figure 1(d)).

### 3.2. Species difference and correlation analysis between HIV group and healthy group

**Figure 2.**
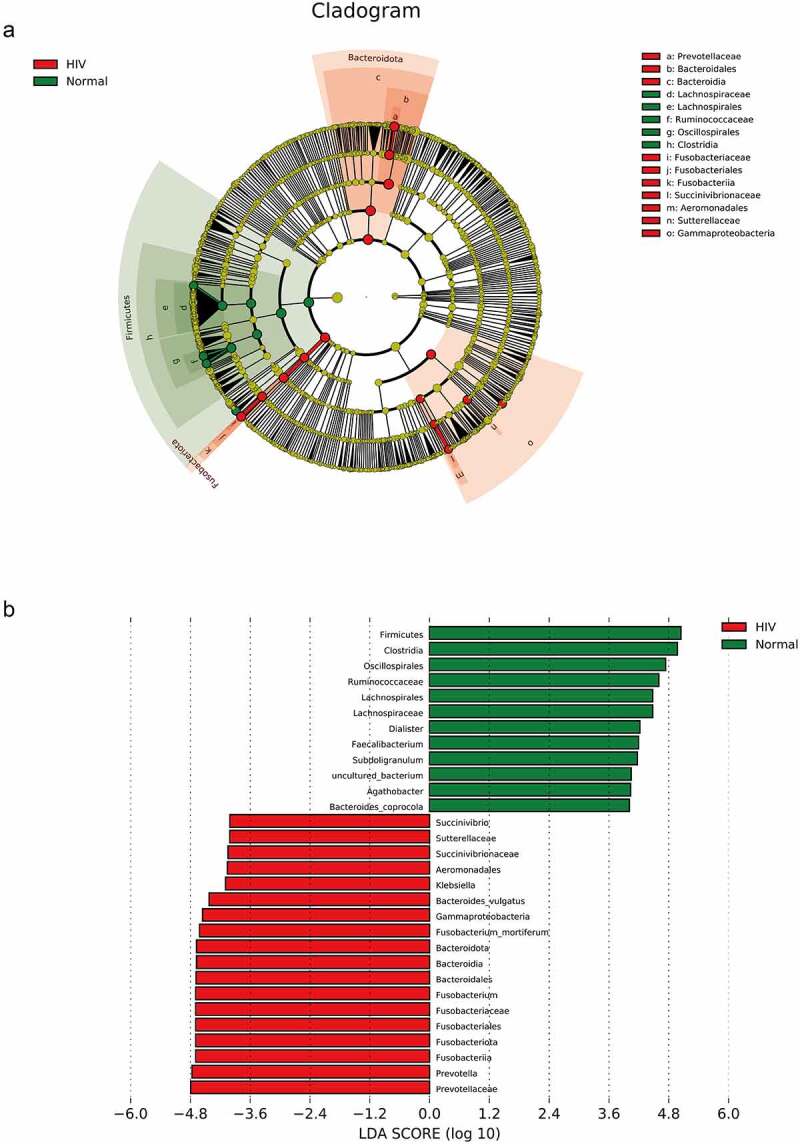
The species difference and association analysis between HIV and healthy groups. The LEfSe analysis was used to estimate the abundance influence of each strain on the difference. A, LEfSe analysis clustering tree. Red represented the HIV group, green represented the healthy group, and nodes of different colors represented the importance of microbiome in the representative group. The yellow nodes indicated the microbiota that did not play an important role in both groups. B, LDA analysis results.

LEfSe analysis was subsequently performed to estimate the abundance influence of each strain on the difference effect, and colonies that produced a significant differential influence on sample classification were identified. Figure 2(a) presented a clustering tree. Red represented the HIV group, green represented the healthy group, nodes of different colors represented the importance of the microbiome in the representative group, and yellow nodes indicated a minor role of microbiota in both groups. The results revealed that from the phylum to family levels, the microbiota with significant differences included Prevotellaceae, Bacteroidales, Bacteroidia, Lachnospiraceae, Lachnospirales, Ruminococcaceae, Oscillospirales, Clostridia, Fusobacteriaceae, Fusobacteriales, Fusobacteriia, Succiniobacteriales, Fusobacteriia, Succiniobacteriales, Fusobacteriia, and Succiniovibrionaceae. Linear regression analysis (LDA) was performed on the microbial groups with significant effects, and the results indicated that at the genus level, there were significant differences in Prevotella, Klebsiella, Fusobacteriota, Fusobacterium, Bacteroides_vulgatus, Succinivibrio, Bacteroidota, Fusobacterium_mortiferum, Subdoligranacteromic, Dialcosister, Bacteroides, Bacteroides Faecalibacterium, uncultured_bacterium, and Agathobacter (Figure 2(b)).

### 3.3. Correlation analysis between differential species and IL-2, IL-8 and TNF-α

**Figure 3.**
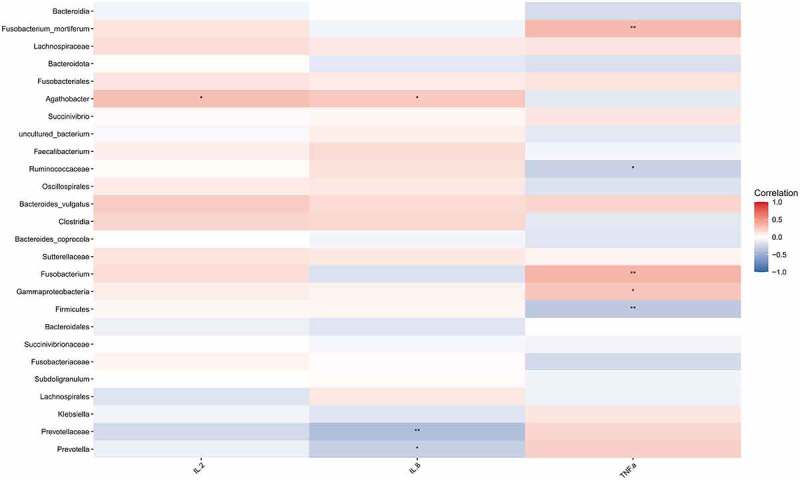
Correlation analysis between different strains and IL-2, IL-8, and TNF-α. Spearman correlation analysis on the different strains and the contents of IL-2, IL-8, and TNF-α between the HIV and healthy groups. Red represented positive correlation and blue represented negative correlation. Asterisk represented a significant difference (p < 0.05).

Subsequently, we performed Spearman correlation analysis on the *differential species* and the contents of IL-2, IL-8, and TNF-α between the HIV and healthy groups. The results were presented in Figure 3, indicating that Fusobacterium_mortiferum, Fusobacterium, and Gammaproteobacteria were positively correlated with TNF-α (p < 0.05), whereas Ruminococcaceae and Bacteroidales were negatively correlated with TNF-α (p < 0.05). Meanwhile, Agathobacter was positively correlated with contents of IL-2 and IL- 8 (p < 0.05), whereas Prevotellaceae, and Prevotella were negatively correlated with IL-8 content (p < 0.05).

### 3.4. The impact of the number difference in CD4 T cells on the intestinal flora of HIV patients

**Figure 4.**
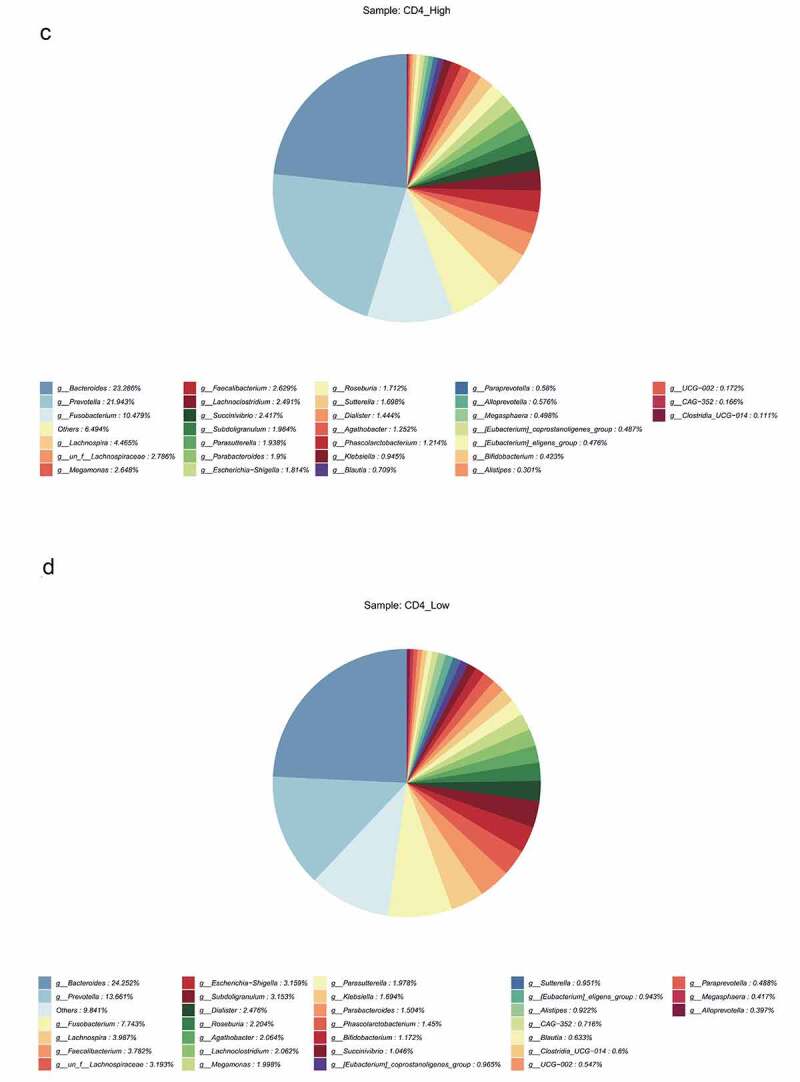
Continued.

The HIV patients were divided into a high CD4 group (≥350/mm^3^) and low CD4 group (<350/mm^3^) according to the number of CD4 T cells, and analyzed with the healthy group, and the differences in flora were compared among the three groups. The results revealed that observed species index, Chao1 index, ACE index, Shannon index, and Simpson index in the high (≥ 350/mm^3^) and low (<350/mm^3^) CD4 groups were markedly lower than those of the healthy group (p < 0.05), but there was no significant difference between the high (≥350/mm^3^) and low (<350/mm^3^) CD4 groups (Figure 4(a)). Species clustering analysis revealed that the top five bacteria in the high CD4 group were Bacteroides (23.286%), Prevotella (21.943%), Fusobacterium (10.479%), Lachnospira (4.465%), and un_f_Lachnospiraceae (2.786%), respectively. And the top five bacteria in the low CD4 group were Bacteroides (24.252%), Prevotella (13.661%), Fusobacterium (7.743%), Lachnospira (3.987%), and Faecalibacterium (3.782%) respectively (Figure 4(b-d)).**Figure 4.**
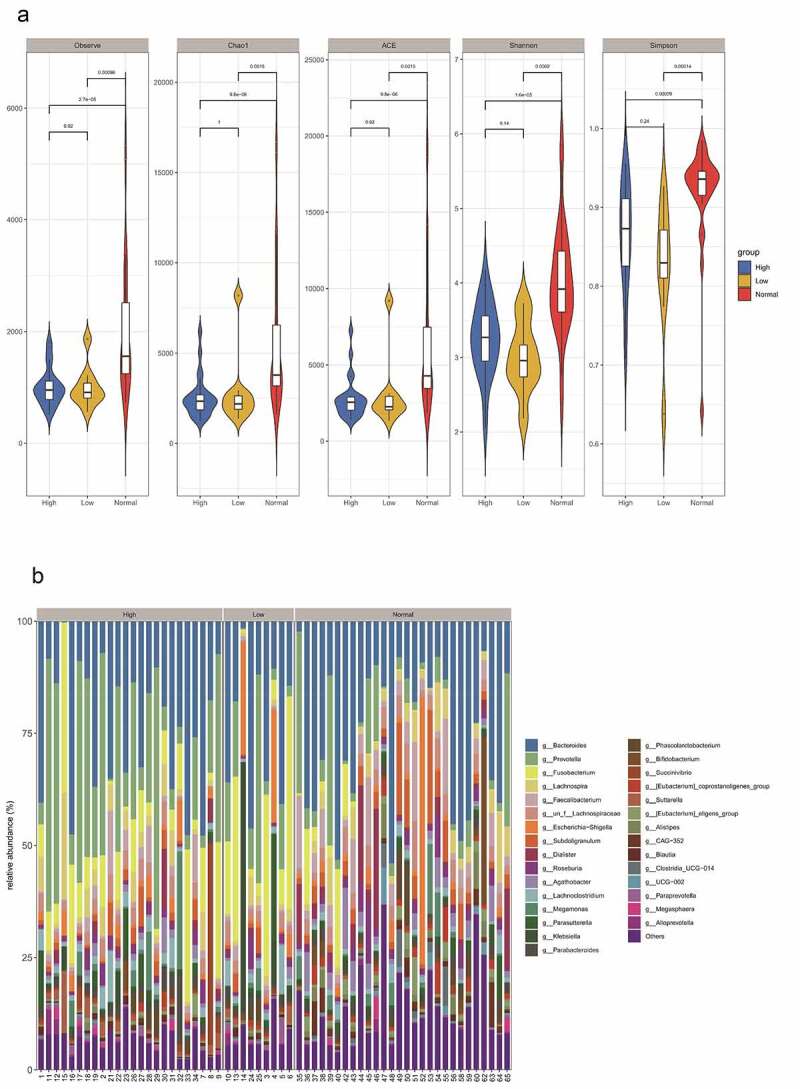
The impact of the difference in CD4 T cell count on the intestinal flora of HIV patients. The HIV patients were divided into a high CD4 group (≥ 350/mm3) and low CD4 group (< 350/mm3) according to CD4 T cell count, and subsequently compared with the healthy group to identify the differences in flora between the three groups. A, Alpha diversity analysis. B, Clustering analysis. C, Bacteria abundance in the high CD4 group. D, Bacteria abundance in the low CD4 group).

**Figure 5.**
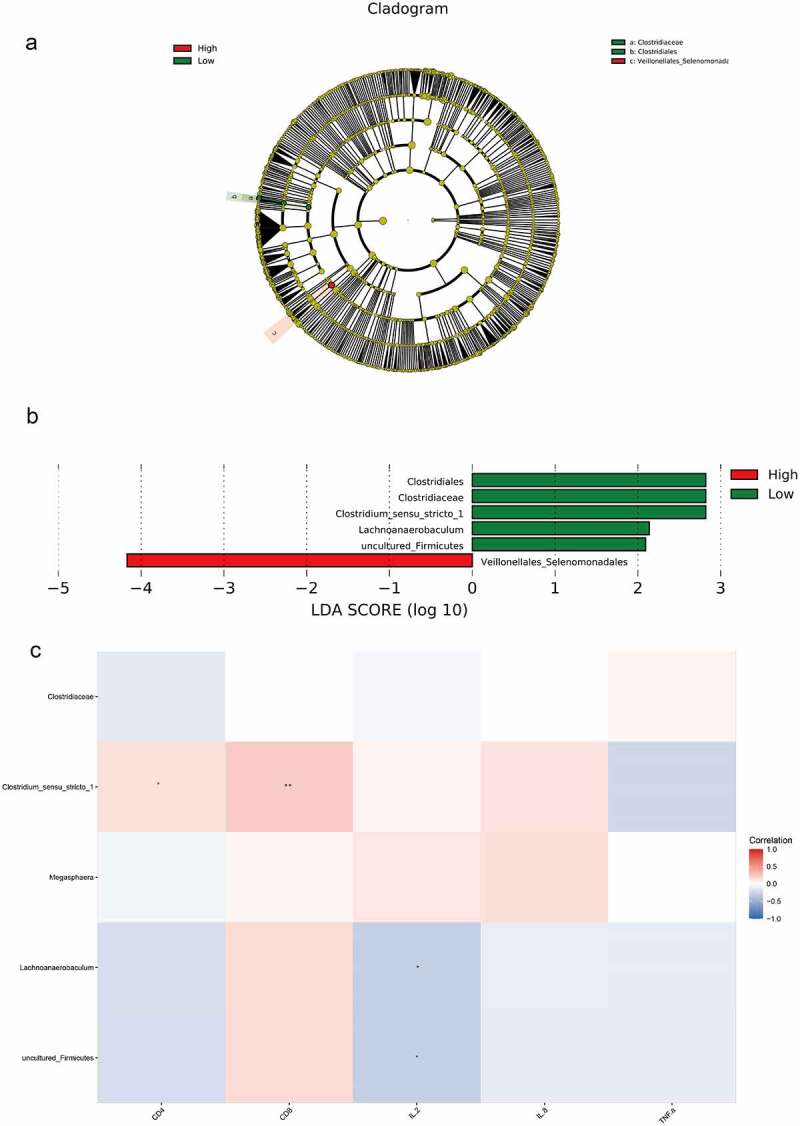
LEfSe analysis and Spearman correlation analysis compared bacteria differences and correlation analysis between the high and low CD4 groups. A, LEfSe analysis of the clustering tree, red represented the high CD4 group, green represented the low CD4 group, and nodes of different colors represented the importance of microbiome in the representative group. The yellow nodes indicated the microbiota that did not play an important role in both groups. B, LDA analysis results. C, Spearman was adopted to analyze the correlation between the differential flora and CD4, CD8, IL-2, IL-8 and TNF-α.

The results of LEfSe analysis revealed that the microbiota with significant differences was Clostridiaceae, Clostridiales, and Veillonellales_Selenomonada in the high and low CD4 groups at the phylum to family levels (Figure 5(a)). Meanwhile, the results of LDA analysis revealed that Clostridium_sensu_stricto_1, Lachnoanaerobaculum, and uncultured_Firmicutes were significantly different at the genus level (Figure 5(b)).

Spearman correlation analysis was used to analyze the correlation between bacterial species and environmental factors in the high and low CD4 groups, and Clostridium_sensu_stricto_1 was positively correlated with the contents of CD4 and CD8 cells, whereas Lachnoanaerobaculum and uncultured_Firmicutes were negatively correlated with IL-2 content (Figure 5(c)).

Collectively, Fusobacterium and Escherichia-Shigella were specific and highly abundant in the HIV group, and Subdoligranulum was specific and highly abundant in the normal population.

## 4. Discussion

HIV-1 infection can injure the gastrointestinal tract, resulting in structural damage to the epithelial barrier and destruction of intestinal homeostasis [4]. Microbial translocation is related to the disease progression of HIV-1 infection. Therefore, the changes of intestinal flora caused by HIV-1 infection and the mechanism of the intestinal flora interaction with mucosal immune cells, allowing to promote inflammatory response, have gained increasing attention all the time. The present study focused on exploring the diversity of intestinal flora, changes in species composition and relative abundance, and the action mechanism of intestinal flora on intestinal immune cells after HIV infection. Additionally, it attempted to offer a comprehensive understanding of the intestinal flora changes in HIV-1 infection and provide a theoretical reference for future AIDS treatment. This study compared and analyzed the differences in the intestinal flora and the changes in CD4 T cells in HIV patients using 16S rDNA sequencing. The results revealed that the abundance of intestinal flora in HIV patients was significantly lower than that of the healthy group. The top five strains in the HIV group were Bacteroides, Prevotella, Fusobacterium, Lachnospira, and Escherichia-Shigella. Fusobacterium_mortiferum, Fusobacterium, and Gammaproteobacteria were positively correlated with TNF-α, whereas Ruminococcaceae, Bacteroidales were negatively correlated with TNF-α. Meanwhile, Agathobacter was positively correlated with contents of IL-2 and IL-8, whereas Prevotellaceae, and Prevotella were negatively correlated with IL-8 content. Furthermore, there was no marked difference in the alpha diversity indices between the high and low CD4 groups among HIV patients. Significant differences in the genus of both the high and low CD4 groups included Clostridium_sensu_stricto_1, Lachnoanaerobaculum, and uncultured_Firmicutes. Clostridium_sensu_stricto_1 was positively correlated with contents of CD4 and CD8 cells, while Lachnoanaerobaculum and uncultured_Firmicutes were negatively correlated with IL-2 content. The present study identified the differentially expressed flora in HIV patients, the high and low CD4 groups, and the association between the differential flora and IL-2, IL- 8, and TNF-α was identified simultaneously.

The research between HIV infection and intestinal flora has a long history. In 2008, Gori et al. reported for the first-time HIV-1 infection that changed the composition of the gut microbiota [17]. Fluorescence in situ hybridization and quantitative PCR were performed to analyze the fecal flora in the HIV-positive and negative control groups. By comparison, the fecal opportunistic pathogens *Pseudomonas aeruginosa* and *Candida albicans* were elevated and bifidobacteria were decreased in the HIV-positive group. A prospective study by Ellis et al. has further revealed that HIV-1 infection is related to the imbalance of intestinal flora [18]. They pioneered the fluorescence quantitative PCR assay to detect the expressions of the 16S rRNA gene and the orders Enterobacter, Bacteroides, and Clostridium in feces [18]. As deep sequencing technology advances, the investigation of the mechanism of intestinal flora in HIV-1 infection is feasible. Changes in the gut microbiota in the distal gastrointestinal tract of adults were found in three dominant phyla Bacteroides, Proteobacteria, and Firmicutes. Generally, the main feature of intestinal flora imbalance is an increased relative abundance of gram-negative bacteria and decreased relative abundance of gram-positive bacteria, especially the immunomodulatory flora [19]. Additionally, some cross-sectional studies have shown that the intestinal flora imbalance remains in the HIV-positive group receiving effective antiviral therapy, and lasts a long time during the treatment process [20]. The results have indicated that antiviral drugs can change the composition of intestinal flora. An overall increase in the relative abundance of Proteobacteria has been detected in the mucosal tissues of both treated and untreated HIV [21,22]. Another study has revealed that the increase of HIV infection associated *Proteobacteria* only occurs in the colon, and no Proteobacteria increase was observed in the feces of the untreated HIV-positive group [5]. In a recent study on the species assessment of bacteria, the relative abundances of *Burkholderia*, *Brachyrhizobium, Acinetobacter*, and *Streptococcus thermophilus* were detected in untreated HIV colonic mucosa. An increase in Desulfovibrio was observed in feces [23,24]. The results of this study found that compared with the samples of the healthy group, at the phylum to family level, the microbiota with significant differences included *Prevotellaceae, Bacteroidales, Bacteroidia, Lachnospiraceae, Lachnospirales, Ruminococcaceae, Oscillospirales, Clostridia, Fusobacteriaceae, Fusobacteriales, Fusobacteriia, Succiniobacteriales, Fusobacteriia, Succiniobacteriales, Fusobacteriia, and Succiniovibrionaceae*. At the genus level, there were significant differences in *Prevotella, Klebsiella, Fusobacteriota, Fusobacterium, Bacteroides_vulgatus, Succinivibrio, Bacteroidota, Fusobacterium_mortiferum, Subdoligranulum, Dialister, Firmicutes, Bacteroides_coprocola, Faecalibacterium*, undoberbacterium, and undoberculture. This is consistent with the results of previous studies, indicating that HIV infection can destroy the ecological structure of intestinal flora [25].

In addition, immune activation and the expression of inflammatory factors are also very important links in HIV research. The analysis of the correlation between intestinal flora and immune activation and inflammatory factors is therefore of vital significance. Multiple research teams have begun to use in vitro models to study the underlying mechanisms that drive microbiota-related immune activation and inflammation in HIV-1 infection. The peripheral blood mononuclear cells of the HIV infection group and the healthy control group were stimulated with Bacteroides, Prevotella, and Veronococcus bacteria lysates, and it was found that the expression levels of TNF-α and IL-10 have significantly increased in the peripheral blood mononuclear cells of the HIV-infected group[26,27] [. Using the human primary intestinal lamina propria monocyte model, the exposure of human primary intestinal lamina propria monocytes to E. coli will enhance HIV-1 infection of CD4 T cells and promote T cell apoptosis in vitro [28]. A low level of intestinal flora in the feces of the HIV-positive group is related to the increased activation level of T cells in the duodenum. In addition, the elevated expression level of Enterobacteriales is related to decreased duodenum CD4 T [29,30]. This study revealed that the microbiota with significant differences was Clostridiaceae, Clostridiales, and Veillonellales_Selenomonada in the high and low CD4 groups at the phylum to family levels. Clostridium_sensu_stricto_1, Lachnoanaerobaculum, and uncultured_Firmicutes were greatly different at the genus level. Spearman correlation analysis was used to analyze the correlation between bacterial species and environmental factors in the high and low CD4 groups, and it was found that Clostridium_sensu_stricto_1 was positively correlated with the contents of CD4 and CD8 cells, whereas Lachnoanaerobaculum and uncultured_Firmicutes were negatively correlated with IL-2 content. The findings are considered to be the highlights of the current study and this study also indicated a correlation between differentially expressed flora and inflammatory factors, expecting to provide a better theoretical basis for future treatment of AIDS.

Although the control of HIV infection and associated morbidity remain to be a major challenge in many parts of the world, the treatment of HIV-positive individuals in developed regions is improved and under a principle of long-term control of multiple comorbidities associated with chronic HIV infection and treatment. Nowadays, HIV microbiome research is still in its infancy, and the mechanism of intestinal flora imbalance needs to be fully explored. As the imbalance of the gut microbial community exacerbates the progression of HIV-1 disease, restoring the ‘normal’ community structure may alleviate the progression of disease in HIV-positive patients. It is therefore that the recognition of host mechanisms, especially inflammatory factors that influence microbial community structure, may bring about certain novel approaches to restore intestinal homeostasis permanently.

## 5. Conclusions

The present study identified the differentially expressed flora between HIV patients and healthy individuals, as well as the high and low CD4 groups. Meanwhile, the association between the differential flora and IL-2, IL-8, and TNF-α was also revealed. Fusobacterium and Escherichia-Shigella were specific and highly abundant in the HIV group, while Subdoligranulum was specific and highly abundant in the healthy group. This research provided some novel considerations for the treatment of AIDS from the perspective of intestinal flora.

In future research, we envisage regulating the balance of the intestinal microbiota of HIV patients (especially those who do not respond to antiretroviral therapy) by supplementing specific probiotics to promote the growth of Th22 cells, Th17 cells, and Tregs cells. The immune function of the intestinal mucosa of HIV patients is expected to be restored to repair the integrity of the intestinal mucosa, thereby preventing the translocation of intestinal microbes and fortifying the number of CD4 cells and their immune function. A complementary treatment method for HIV patients is expected to be available for delaying the onset or cure of AIDS.

## Highlights

- Fusobacterium and Escherichia-Shigella are highly abundant in the HIV group.
- The differential flora is related to the expressions of IL-2, IL-8, and TNF-α.
- It provides a better theoretical basis for future treatment of AIDS.
